# Maturation arrest of human oocytes at germinal vesicle stage

**DOI:** 10.4103/0974-1208.74161

**Published:** 2010

**Authors:** Zhi Qin Chen, Teng Xiao Ming, Hans Ingolf Nielsen

**Affiliations:** Department of Assisted Reproduction, Shanghai First Maternity and Infant Hospital, Tong Ji University, China

**Keywords:** GV block, infertility, IVF, meiosis, oocyte maturation arrest

## Abstract

Maturation arrest of human oocytes may occur at various stages of the cell cycle. A total failure of human oocytes to complete meiosis is rarely observed during assisted conception cycles. We describe here a case of infertile couples for whom all oocytes repeatedly failed to mature at germinal vesicle (GV) stage during *in vitro* fertilization/Intra cytoplasmic sperm injection (IVF/ICSI). The patient underwent controlled ovarian stimulation followed by oocyte retrieval and IVF/ICSI. The oocytes were stripped off cumulus cells prior to the ICSI procedure and their maturity status was defined. The oocyte maturation was repeatedly arrested at the GV. Oocyte maturation arrest may be the cause of infertility in this couple. The recognition of oocyte maturation arrest as a specific medical condition may contribute to the characterization of the currently known as “oocyte factor.” The cellular and genetic mechanisms causing oocyte maturation arrest should be the subject for further investigation.

## INTRODUCTION

In the human ovary, each fully grown oocyte resumes maturation in response to gonadotrophins. This process is completed after oocytes reach metaphase II (MII) stage. After oocytes begin to mature, their nuclei–germinal vesicles (GV) break down and chromosomes condense (germinal vesicle breakdown, GVBD). Chromosomes are then arranged in MI stage which is followed by anaphase I to telophase I transition and oocytes are arrested in MII, ready for fertilization[1]

Women undergoing controlled ovarian hyperstimulation (COH) prior to IVF are treated by various protocols aimed at inducing multiple follicular growth. Oocyte meiotic maturation is induced by hCG, acting as a surrogate LH surge. Following the LH surge, the resumption of meiosis and achievement of second metaphase occurs within 18 h and 28-38 h, respectively.[2] Normally, in conventional protocols by the time of retrieval, the majority oocytes have completed their maturation and are collected at the metaphase II (MII) phase. Although it is common for a few oocytes to remain immature despite ovarian stimulation and human chorionic gonadotrophin (hCG) administration,[3] the complete failure of all oocytes at GV stage to mature *in vivo* is extremely rare, and only a few of such cases have been described in the literature.[4,8]

We report a case of primary infertility in which two consecutive IVF attempts yielded oocytes with the evidence of complete oocyte maturation arrest at GV stages of the cell cycle and as a consequence of no opportunity for fertilization.

## CASE REPORT

A 31-year-old woman with a 7-year history of primary infertility was referred to our IVF clinic for unexplained infertility. She underwent a thorough infertility investigation without any abnormal findings identified. She had regular ovulatory cycles and failed to conceive after at least six cycles of ovulation induction, and four cycles of intrauterine insemination (IUI). She was in good general health, was a non-smoker, and with no family history of infertility. No history of exposure to any medications for other medical conditions during or prior to IVF, she also did not have possible past exposure to environmental and occupational toxicants and excessive X-ray irradiation. She exhibited a normal female (46, XX) karyotype. She had normal day 3 FSH levels (5.68 IU/l) and a normal response to both induction of ovulation and ovarian stimulation. Her husband’s sperm were as follows: Volume- 5 ml, Count: 41 mill/ml, Motility: RL-68%, SL-13.93, NP-10.92, IM-7.15, morphology: normal

The patient was treated according to our routine IVF treatment protocols. In short, the patient was pre-treated with Dipherline 1.25 mg (Ipsen Pharma Biotech, France) i.m. at the mid-luteal phase of the cycle preceding the treatment cycle and received Puregon (Organon, Holland) for ovarian stimulation. Ovarian response was monitored by serial transvaginal scanning and hCG (lizhu, China) 10000 IU i.m. was given when there were at least 3 follicles >16 mm in diameter. The serum E2 concentration was measured on the day of hCG administration. Oocyte retrieval was scheduled 36h after the hCG injection. In the second attempt, human menopausal gonadotrophin (HMG, lebaode, lizhu, China) was used for controlled ovarian stimulation and Recagon hCG (Ovidrel Serono, Switzerland) in dose of 250 ug subcutaneously for final oocyte maturation [Table 1].

**Table 1:** Gonadotrophin stimulation and follicular responses for the two seperate cycles presented

Cycle	Protocol	Days of FSH stimulation	Drugs (Total IU of FSH/HMG	LH on day of hCG (IU/L)	E2 on day of hCG (pg/ml)	Dose of hCG	No. of oocyte pick up	Maturation status
1	Long	12	Puregon (2300IU) / HMG (375IU)	0.97	3425	10000 IU	17	GV
2	Long	11	HMG (2400IU)	1.40	5369	250 ug (Ovidrel)	12	GV

**Figure 1:**
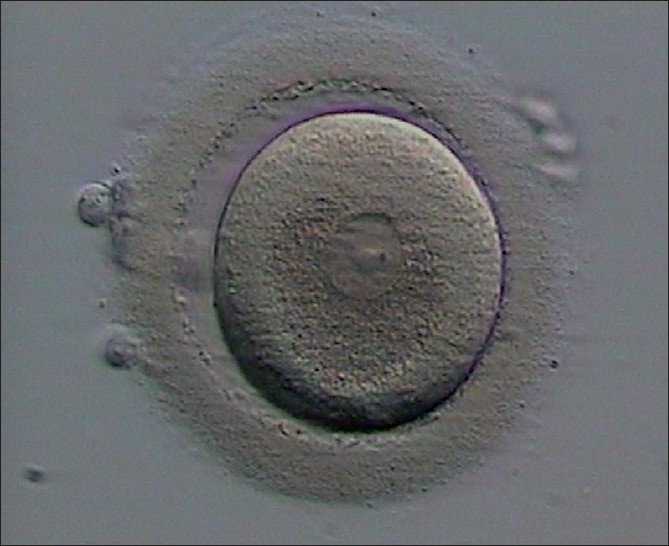
One of the 17 oocytes at the first trial remained at the germinal vesicle (GV) stage after being stripped of cumulus cells for the ICSI procedure (day 0), and after 24-48 h of culture. (day 1 and day 2), its maturity status was defined the same (GV), arrow shows germinal vesicle

Oocytes were retrieved 36-38 h after hCG administration. Following retrieval, the oocytes were stripped off cumulus cells for the ICSI procedure and their maturity status was defined. All the oocytes were GVC therefore the oocytes were further cultured for 3 days [Figure 1].

**Figure 2:**
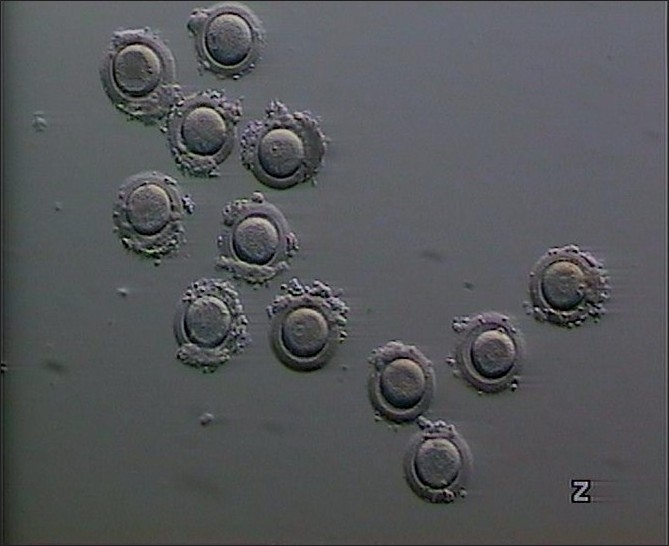
12 oocytes were aspirated at the second attempt, all of them were at the GV stage, and failed to proceed in meiosis beyond this stage even after extended culture

On her first IVF attempt, 17 oocytes that were retrieved remained at the germinal vesicle (GV) stage, even after 48 h of culture. On the second attempt, we prolonged the hCG–oocyte retrieval interval to 38 h; again 12 oocytes were aspirated, all of them at the GV stage, and failed to proceed in meiosis beyond this stage even after extended culture [Figure 2].

## DISCUSSION

Approximately 20%–30% of oocytes collected for IVF are meiotically immature at the time of oocyte retrieval,[9] and this is probably because of the stimulation of multiple follicles. When the percentage of incompetent oocytes was ≥25%, most of the IVF outcomes were markedly reduced.[3] It is common for a small percentage of oocytes to arrest at the GV or MI stage, however, it is extremely rare for complete oocyte maturation failure to occur in IVF treatment and only a few cases have been reported previously.[4–8] Oocytes from some patients were collected at GV stage and did not resume meiosis when cultured *in vitro*.[6,8] In some other cases, oocytes were collected in MI and were unable to complete meiosis up to MII.[7] Moreover, in some patients, oocytes did not respond properly to fertilizing sperm.[5,10]

The reason for the the oocyte maturation arrest is not clearly understood. Like the cases reported earlier, this patient exhibited the same phenomena in two separate occasions, and the same pattern of maturation arrest was repeatedly observed. Thus, for oocyte maturation arrest is not a sporadic event of abnormal response to ovarian stimulation or poor culture conditions in a certain cycle or laboratory. Failure to resume meiosis *in vivo* may arise at one of the following three levels: (i) absent or incomplete LH surge; (ii) derangements in the signaling mechanism from the surrounding cumulus cells; and (iii) intrinsic oocyte factors.

*In vivo*, abnormal or insufficient LH surge may interfere with progression of meiosis by one of the following mechanisms- incorrect timing of the hCG injection, lack of LH activity (hCG batch problem, i.e. inactive isoform), disturbed hormone delivery or dysfunctional LH receptors. All of the above theories seem unlikely in our case in view of the repetitive nature of this phenomenon, despite use of different dose and type of hCG,. At the first attempt oocytes were retrieved 36 h after hCG administration, and at the second attempt, we prolonged the hCG–oocyte retrieval interval to 38 h. The patient also exhibited normal ovarian steroidogenesis (E2 output) before and at the time of hCG administration, and there was no difficulty at oocyte retrieval.

*In vivo*, derangements in the signaling mechanism from the surrounding cumulus cells may account for meiotic maturation arrest in some of the cases.[6,7] Follicular somatic cells may suppress the expression of meiotic competence in oocytes *in vivo*. It has been recently demonstrated in the mouse that follicles lacking the gap junction protein connexin43 failed to develop a multilaminar granulosa cell layer, and exhibited retarded oocyte growth.[11] In this case, apparently mature cumulus cells with normal appearance surrounded the oocytes were observed. We were unsuccessful in our attempt to induce germinal vesicle breakdown (GVBD) in these oocytes *in vitro* after extended culture.

Like us, others have also failed to induce completion of meiosis in cases with oocyte maturation arrest at the GV stage. In the case reported by Rudak *et al*., extended incubation *in vitro* for several days failed to induce oocyte maturation beyond the GV stage.[4] Hartshorne *et al*., have noted variable degrees of cumulus expansion following oocyte retrieval in a case with meiotic arrest at the GV stage.[6] They were able to induce expansion of cumuli after exposure to FSH and hCG *in vitro*, suggesting that the cumulus cells were capable of responding to gonadotrophins, but their response was not translated to the oocyte or that the oocyte was unable to respond to the signals from the cumulus. In this case, no advance in meiotic stage was observed following removal of the cumulus cells. Thus, although the possibility of abnormal signaling between the cumulus cells and the oocytes cannot be completely excluded

It has been demonstrated that oocytes may become competent to resume meiosis only after they have acquired a certain size. Growing oocytes are yet unable to respond to maturation signals both *in vivo* and *in vitro* and remain arrested in the diplotene stage. Those oocytes (<60 mm) not attaining their full size are unable to undergo GVBD and remain arrested at this stage or eventually they may mature only to MI. Only fully-grown oocytes respond to gonadotrophic signals and mature to MII oocytes in pre-ovulatory follicles. In our case, the oocytes appeared to be normal in size as expected for mature oocytes, although specific measurements were not made. Therefore, it seems unlikely that disturbed oocyte growth was the cause of meiotic incompetence.

A study on pig and cattle shows that the ability to initiate maturation is related to the follicle size from which the oocyte is collected.[12] In humans, it remains a question if, in some rare cases, the follicular growth is not accompanied with relevant oocyte growth. Thus, the follicle would attain the appropriate size at the time of aspiration while the oocyte is still slightly developmentally behind.

In the case reported by Hartshorne *et al*., the nuclei of GV-arrested oocytes were examined by fluorescence microscopy following chromatin staining.[6] The appearance of the chromatin in all oocytes was consistent with initiation of meiotic progression from prophase I towards MI, characteristic of arrest at entry to M-phase of the cell cycle. It has been shown that chromatin configurations differ in immature human oocytes as compared to large antral oocytes. It is assumed that only those oocytes in which the nucleolus is surrounded with a ring of condensed chromatin mature better and are more developmentally competent after fertilization.[13–15]

Fully grown mammalian oocytes are arrested at two points of maturation. The first point of arrest is at the GV stage when oocytes are awaiting the gonadotrophin signal or the release from an inhibitory follicular environment. The second point is at MII stage when oocytes are waiting for fertilization.[16] The capacity to resume and complete meiotic maturation is probably acquired during oogenesis. This capability is known as meiotic competence acquisition. The process of maturation is under the control of the maturation promoting factor (MPF). In immature oocytes, MPF is present in an inactive phosphorylated form as a complex of Cdk 1(p34cdc2) and cyclin B. This phosphorylation is controlled by Myt1 kinase. The dephosphorylation of MPF is induced by Cdc25 phosphatase (probably by Cdc25B). The activity of MPF reaches its peak in MI oocytes and then decreases during the anaphase to telophase transition. Thereafter, high levels of MPF are again restored and oocytes are kept at this stage under the influence of a cytostatic factor (CSF).[17] MPF is fully degraded when oocytes are fertilized.[18,19]

The spontaneous resumption of meiosis is believed to be triggered by a fall in oocyte levels of cyclic AMP (cAMP).[20,21] *In vivo* meiotic arrest is achieved by a stimulatory G protein (Gs) acting on adenylyl cyclase,[22] and this activity cannot be maintained when oocytes are released from their intrafollicular environment. The decrease in intra-oocyte cAMP directly or indirectly activates MPF. The most likely mechanism by which cAMP maintains arrest has been studied in frog oocytes. It is through activation of protein kinase A(PKA), which in turn phosphorylates CDC25[23]. Switching on of MPF is further governed by the balance in the regulatory activity of Wee1/Myt1 kinases, which cause an inhibitory phosphorylation of CDK1 at Thr14 and Tyr15 and hold the heterodimer in an inactive state (so-called pre-MPF); The CDC25 phosphatases also cause an activating dephosphorylation of CDK1 at the same sites. High CDC25 and low Wee1/Myt1 activity are needed for switching on the CDK1 component of MPF. Phosphorylated CDC25 is further sequestered in the cytoplasm by 14-3-3, a family of small acidic proteins, preventing its accumulation in the nucleus.[24] Thus, alterations in spatial and temporally MPF is regulated spatially (nucleus versus cytoplasm) and temporally (CDC25 and Wee1 kinases) will underlie the phenomenon of oocyte competency.

Levran *et al*., (2002),[10] reported that the inability of oocytes to mature was observed repeatedly, in patients with rare heritable molecular defects that are responsible for the inability of these oocytes to initiate the activation of MPF. It is impossible to define these defects precisely but a paper published by Lincoln *et al*., (2002)[25] showed that this possibility may theoretically exist. These authors generated Cdc25B–/– knockout mice and found that oocytes from these females were ovulated at GV stage and when further cultured *in vitro* were unable to undergo GVBD and remained GV stage-arrested. The wild type Cdc25B mRNA microinjection into these oocytes triggers the resumption of meiosis. This experiment shows that Cdc25B may play an important role during oocyte maturation. The process of maturation, however, is much more complex and not yet fully understood.[26] In somatic cells, the transition from one stage to another one is perfectly controlled by so-called checkpoint controls. It is unclear whether equivalent control mechanisms also exist in mammalian oocytes.[27] We may suppose that the same, or similar, cell cycle control mechanisms regulate maturation of human oocytes.[28]

We cannot suggest a therapeutic approach that would help to overcome the blocks in oocyte maturation and sustain successful IVF. Extending the hCG to retrieval interval, increasing the hCG dose, and extended *in-vitro* culture all failed to overcome the meiotic arrest. Additional therapeutic approaches could include *in-vitro* maturation (IVM) of immature oocytes, extended culture in media enriched with yet undetermined factors necessary for oocyte maturation, and intracytoplasmic injection of donor cytoplasm or maturation promoting factors. All of these interventions should be currently regarded as speculative with uncertain effect, and the progress in management of these difficult cases has to await better understanding and knowledge of the processes manipulating oocyte maturation. Currently, oocyte donation seems to be the most viable option. At times, many patients still can’t accept this option due to emotional and religious considerations. More information on the physiology of oocyte maturation is needed before the exact nature of the defects involved in achievement of meiotic competence can be determined and effective therapy can be adopted.

## CONCLUSION

Defective LH surge, disturbed cumulus–oocyte interaction and abnormal oocyte growth seem an unlikely reason for maturation arrest in our case. The possibility of an intrinsic oocyte defect remains the most appropriate probability cause for option as the cause of oocyte maturation arrest. For our debate in the article, we do believe that these data may, at least partially, shed some light on the meiotic arrest problem in assisted human reproduction.
